# MicroRNA-630 suppresses tumor metastasis through the TGF-β- miR-630-Slug signaling pathway and correlates inversely with poor prognosis in hepatocellular carcinoma

**DOI:** 10.18632/oncotarget.8047

**Published:** 2016-03-14

**Authors:** Wei-xun Chen, Zhan-guo Zhang, Ze-yang Ding, Hui-fang Liang, Jia Song, Xiao-long Tan, Jing-jing Wu, Guang-zhen Li, Zhuo Zeng, Bi-xiang Zhang, Xiao-ping Chen

**Affiliations:** ^1^ Hepatic Surgery Centre, Tongji Hospital, Tongji Medical College, Huazhong University of Science and Technology, Wuhan, 430030, China

**Keywords:** MiR-630, microRNA, HCC, EMT, TGF-β

## Abstract

The epithelial-mesenchymal transition (EMT) is the key process that drives tumor metastasis. Accumulating evidence suggests that the deregulation of some microRNAs (miRNAs), is implicated in this process. Here, we highlight the function and molecular mechanism of miR-630 and its potential clinical application in hepatocellular carcinoma (HCC). First, we identified the clinical relevance of miR-630 expression in a screen of 97 HCC patient tissues. Patients with low miR-630 expression had higher recurrence rates and shorter overall survival than those with high miR-630 expression. Functional studies demonstrated the overexpression of miR-630 in HCC cells attenuated the EMT phenotype *in vitro.* Conversely, inhibition of miR-630 promoted EMT in HCC cells. Mechanistically, our data revealed that miR-630 suppressed EMT by targeting Slug. Knockdown of Slug expression reversed miR-630 inhibitor-mediated EMT progression. Furthermore, we found that the TGF-β-Erk/SP1 and JNK/c-Jun signaling pathways repressed miR-630 transcription through occupying transcription factor binding sites. Ectopic expression of miR-630 restored the TGF-β-activated EMT process. Taken together, these findings demonstrate, in HCC cells, miR-630 exerts its tumor-suppressor functions through the TGF-β-miR-630-Slug axis and provides a potential prognostic predictor for HCC patients.

## INTRODUCTION

Hepatocellular carcinoma (HCC) is the third leading cause of cancer mortality worldwide [1, 2]. Most patients with HCC are diagnosed at an advanced stage that renders therapy ineffective. Although numerous advanced therapeutic strategies, such as hepatic resection or liver transplantation, have been used in recent years, the five-year overall survival rate is only 30% [3]. Frequent intra-hepatic and distant metastases lead to a high cancer recurrence rate and poor overall survival [4]. Thus, the molecular mechanisms governing prognosis need to be understood urgently.

MiRNAs are a class of single-stranded, non-coding endogenous RNAs that inhibit gene expression at the post-transcriptional level by impeding mRNA translation or degrading target mRNAs [5]. Accumulating evidence has shown that miRNA dysfunction plays an important role in many diseases, including hepatic carcinogenesis. They function as oncogenes and contribute to proliferation and metastasis in HCC [6–8]. Recently, miR-630 has been reported to exert pleiotropic functions and affect proliferation, metastasis, and apoptosis, thereby acting as either an oncogene or a tumor suppressor in different cellular contexts. Through targeting the oncogenes LMO3, metadherin, CDC7 kinase, and IGF-1R, miR-630 has been demonstrated to be a tumor suppressor gene that represses cancer cell proliferation and metastasis or induces apoptosis and death in lung, breast, and pancreatic cancers [9–13]. By contrast, miR-630 acts as an oncogene in ovarian, colorectal, gastric, and renal cell carcinomas [14–18]. To date, the clinicopathological and prognostic value of miR-630, its regulatory networks, and mechanism of deregulation in HCC remain elusive. Here, we investigated the biological functions and the underlying molecular mechanisms of miR-630 in human HCC.

Using human HCC tissue specimens, we found that miR-630 was inversely correlated with tumor metastasis and clinicopathological stage. Forced expression of miR-630 repressed EMT and knockdown of miR-630 increased metastasis of HCC by targeting Slug *in vitro* and *in vivo*. Furthermore, miR-630 transcription could be inhibited by TGF-β-Erk/SP1 and JNK/c-Jun signaling pathways. Thus, the TGF-β-miR-630-Slug axis may provide a potential miRNA-based therapy for preventing HCC metastasis.

## RESULTS

### Decreased miR-630 expression in HCC is associated with metastasis and poor clinical outcomes

**Figure 1:**
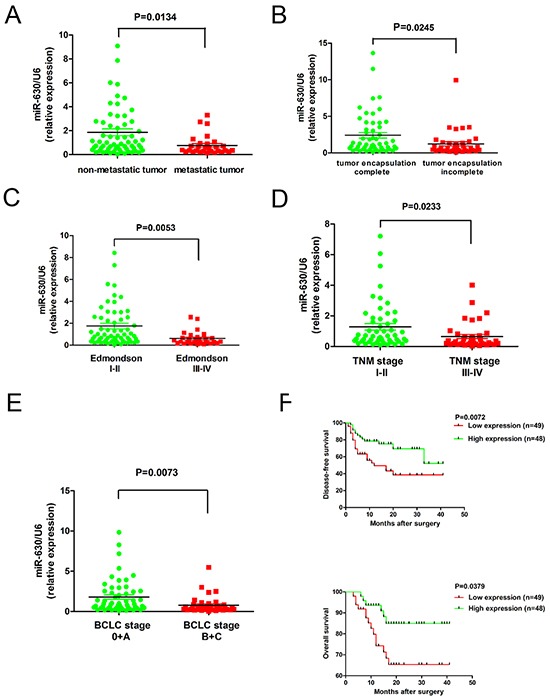
The down-regulation of miR-630 in HCC is associated with metastasis and poor clinical outcomes **A.** The expression of miR-630 in 97 liver tumors was quantified using RT-PCR. P-values correspond to the comparison of miR-630 expression between metastatic and non-metastatic HCC tissues and statistically significant differences are denoted by a P<0.05. **B.** Expression of miR-630 in incompletely encapsulated HCC tumors is lower than that of completely encapsulated tumors. **C.** Edmondson-Steiner tumor grades I-II and III-IV **D.** TNM stage I-II and III-IV **E.** BCLC stage 0+A and B+C **F.** Kaplan–Meier curves of the relationship between miR-630 expression and disease-free and overall survival.

To investigate the clinical significance of miR-630 expression in HCC, we analyzed 97 tumor tissues using quantitative real-time PCR. Compared with non-metastatic tumor tissues, the relative expression of miR-630 was significantly reduced in metastatic tumor tissues (*P=0.0134*, Figure 1A). Furthermore, patients with incomplete encapsulation of their tumors had a significantly lower miR-630 expression compared with patients with completely encapsulated tumors (*P=0.0245*, Figure 1B). Moreover, we observed that the miR-630 expression level was inversely associated with the tumor Edmondson-Steiner stage (I-II/III-IV) (*P=0.0053*, Figure 1C), tumor-node-metastasis (TNM) stage (I-II/III-IV) (*P=0.0233*, Figure 1D) and Barcelona-Clinic Liver Cancer (BCLC) stage (0+A/ B+C) (*P=0.0073*, Figure 1E). To determine the relationship between miR-630 expression levels and clinicopathological features, the 97 patients in the study were divided into two groups according to the median level of miR-630 expression among them. High miR-630 levels were negatively associated with AFP (*P=0.003*), tumor number (*P=0.028*), vascular invasion (*P=0.015*), Edmondson-Steiner stage (*P=0.007*) and BCLC stage (*P=0.002*; Supplementary Table S1) but not tumor size. Kaplan-Meier curves showed that patients with low miR-630 expression had a higher recurrence rate (*P=0.0072*) and shorter overall survival (OS) (*P=0.0379*) compared with patients with high miR-630 expression (Figure 1F). However, clinicopathological features were not correlated with disease-free survival as determined by multivariate analysis (Supplementary Table S2). In summary, decreased miR-630 expression levels correlate with poor HCC prognosis, suggesting that inhibition of miR-630 expression may contribute to the progression of HCC.

### MiR-630 inhibits HCC cells migration, invasion, and EMT *in vitro*

**Figure 2:**
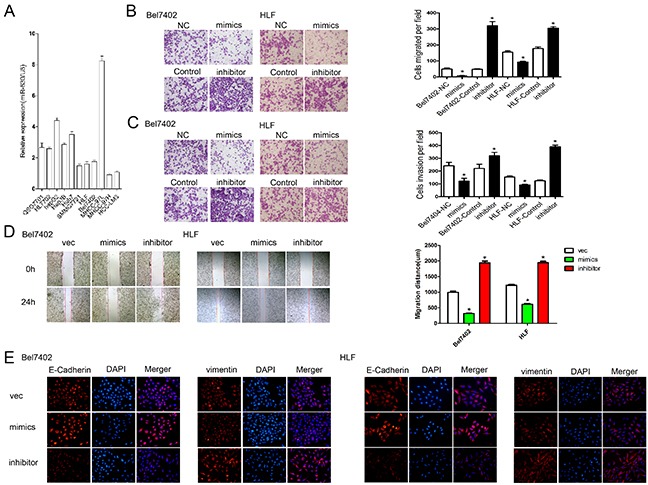
miR-630 inhibits migration, invasion, and EMT in HCC **A.** The expression of miR-630 in different liver and HCC cell lines was determined by qRT-PCR and normalized against U6 expression (endogenous control). **B.** Migration of Bel7402 and HLF cells as measured by the Transwell assay. Representative photographs were taken at a magnification of ×100. **C.** Invasion of Bel7402 and HLF cells as measured by the Transwell assay. Representative photographs were taken at a magnification of ×100. **D.** The wound healing assay was used to assess HCC cell migration. A confluent monolayer of HCC cell was transfected with vector, mimics or inhibitor. Photographs were taken immediately (0 h) after the scratch was made and at 24 h after wounding. The amount of wound closure that occurred after 24 h was quantified. **E.** Immunofluorescent staining of Bel7402 and HLF cells. DAPI was used to show the location of the nuclei (blue). Representative photographs of immunofluorescence were taken at a magnification of ×200.

To investigate the role of miR-630 in HCC progression, we measured the miR-630 expression in normal human liver cell lines (QSG7701, HL7702) and HCC cell lines with different metastatic potentials (HCCLM3, MHCC97H, MHCC97L, SMMC-7721, HLF, Bel7402, HepG2, Hep3B, Huh7) [19] (Figure 2A). The expression of miR-630 in the highly metastatic cell lines (HCCLM3 and MHCC97H) was lower than those in the low-metastatic cell lines (SMMC-7721, HLF, Bel7402, MHCC97L), but unchanged between the non-metastatic cell lines (HepG2, Hep3B, Huh7) and the normal cell lines (QSG7701, HL7702). We selected two HCC cell lines, Bel7402 and HLF, for further study, as they had the median level of miR-630 expression out of all the cell lines. To explore the role of miR-630 in HCC cells, Bel7402 and HLF were transfected with miR-630 mimics or inhibitors (Supplementary Figure S1A). Neither the overexpression nor inhibition of miR-630 altered cell growth in either cell lines (Supplementary Figure S1B). Furthermore, the Transwell assays with and without Matrigel showed that ectopic expression of miR-630 significantly inhibited the migration and invasion of Bel7402 and HLF cells. In contrast, the migration and invasion rates increased when endogenous miR-630 was silenced with miR-630 specific inhibitors (Figure 2B, 2C). The wound healing assay also indicated that up-regulation of miR-630 significantly suppressed cell migration while, decrease in miR-630 leads to a significant increase in cell migration (Figure 2D). These data provide evidence that miR-630 suppresses HCC cell migration and invasion but not proliferation *in vitro*. EMT plays a critical role in promoting migration and invasion, so we speculated that suppression of migration and invasion by miR-630 might also impact EMT. To investigate this hypothesis, we examined the expression of the epithelial makers E-cadherin, as well as the mesenchymal maker vimentin. Immunofluorescent staining of cells transfected with miR-630 mimics showed an increase in E-Cadherin and a decrease in vimentin, whereas we found the opposite with miR-630 inhibitors (Figure 2E). These results suggest that miR-630 can reverse EMT in HCC cells.

### Reduction of miR-630 expression increases HCC metastasis *in vivo*

**Figure 3:**
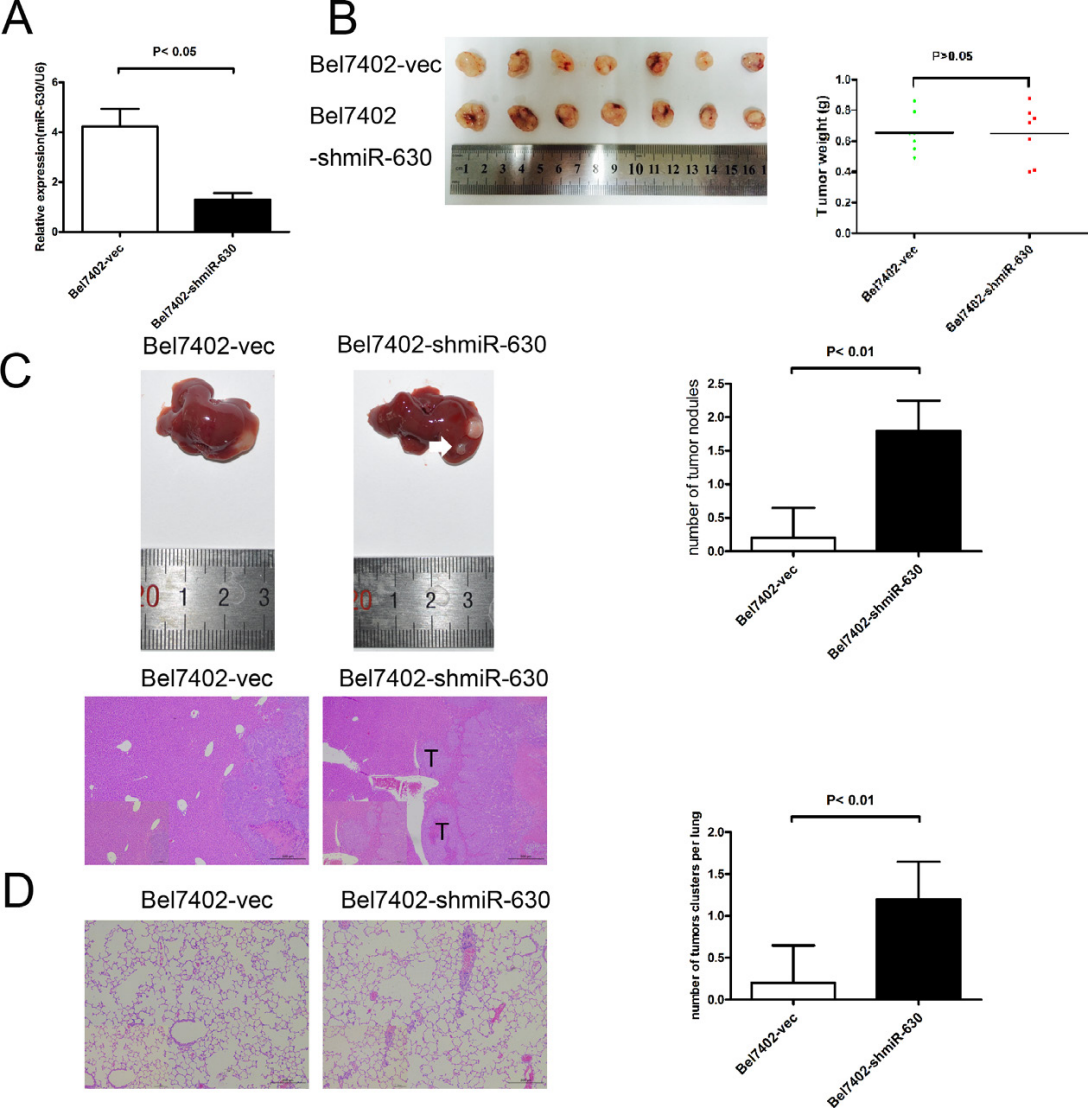
miR-630 reduces metastasis of HCC cells **A.** Relative expression of miR-630 detected by RT-PCR in two HCC cell lines stably transfected with shmiR-630 or miR-NC. **B.** Mean tumor weight was measured on the indicated days. **C.** Representative images of liver hematoxylin and eosin (H&E) sections (lower panel) and statistical analysis of tumor numbers are shown (n=5, upper panel). T, tumor. Local metastasis lesions were measured after eight weeks. White arrows indicate local metastatic foci. Original magnification was ×40. **D.** Representative H&E staining of mice lungs from the different groups is shown. Original magnification was ×100. Statistical analysis of tumor numbers is shown (n=5).

To confirm the effects of miR-630 expression *in vivo*, Bel7402-shmiR-630 cells were constructed (Figure 3A). Bel7402-shmiR-630 and Bel7402-vec cells were injected subcutaneously into nude mice. The tumor weights of mice in the Bel7402-shmiR-630 group were not statistically different compared with those of the Bel7402-miR-vec group (Figure 3B). To further investigate the effects of miR-630 on tumor metastasis *in vivo*, orthotopic mouse models were established by transplanting Bel7402-shmiR-630 and Bel7402-miR-vec tissue into nude mice. Eight weeks post-transplantation, the mice xenografted with Bel7402-shmiR-630 tissue had a significant increase in the number of intra-hepatic lesions and lung metastatic nodules compared with the control group (Figure 3C, 3D). These results indicate that miR-630 functions as a tumor suppressor in HCC by suppressing metastatic colonization but not tumorigenesis.

### MiR-630 directly suppresses Slug and regulates the expression of EMT-related markers in HCC

**Figure 4:**
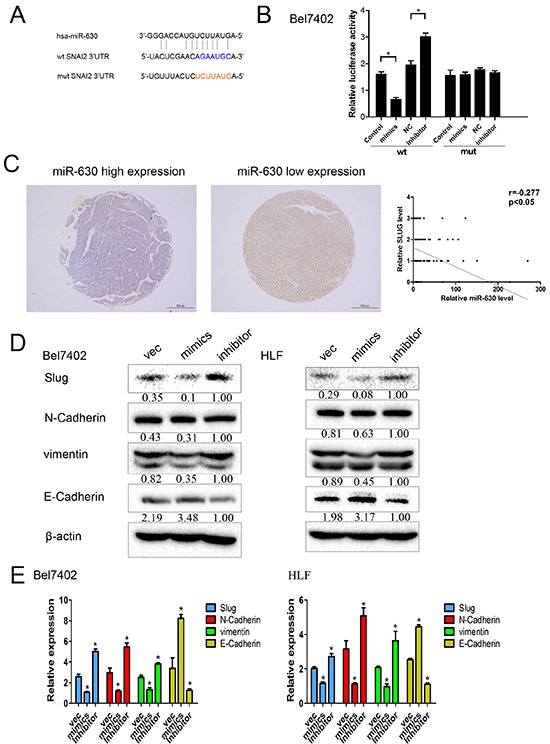
miR-630 directly targets Slug and EMT-related markers in HCC cells **A.** miR-630 and its putative binding sequence in the 3′-UTR of Slug. The mutant miR-630 binding site was generated in the complementary site for the seed region of miR-630 (WT, wild type; MUT, mutant). **B.** Bel7402 cells were co-transfected with a psiCHECK-2 construct containing either the WT or MUT 3′-UTR region of Slug and miR-630 mimics or inhibitor, respectively. The luciferase reporter assay was used 24 h post-transfection to assess transcriptional activity. Relative luciferase activity was plotted as the mean ± SEM of three independent experiments. **C.** Immunostaining of Slug was performed in 80 HCC tissues. The correlation of miR-630 expression with Slug expression was analyzed. The expression of miR-630 was detected via qRT-PCR, and the expression of Slug was detected via immunohistochemical staining. **D.** Western blot analysis of Slug and EMT-related markers expression. β-actin was included as a loading control for each sample. **E.** qRT-PCR analysis of the expression levels of Slug and EMT-related markers. GAPDH was included as a loading control for each sample.

To further elucidate the underlying molecular mechanism by which miR-630 suppresses HCC metastasis, we used publicly available databases, including TargetScan (http://www.targetscan.org/) and PicTar (http://pictar.mdc-berlin.de/) to search for the genes targeted by miR-630 and identified Slug (SNAI2) as one of the target genes (Figure 4A). Though Slug expression has been shown in lung cancer cells [18], we explored whether miR-630 decreased its expression in HCC cells. Luciferase Reporter assays showed that the activity of luciferase linked with the 3′UTR of Slug was suppressed in Bel7402 cells transfected with miR-630 mimics compared with control cells. Conversely, miR-630 inhibition caused a significant increase in luciferase activity (Figure 4B). Furthermore, Slug expression was inversely correlated with miR-630 expression in 80 HCC tumor tissues as determined by Spearman's correlation analysis (Figure 4C). Western blot (WB) and RT-PCR analysis consistently indicated that the expression levels of Slug, N-Cadherin and vimentin were reduced in miR-630-overexpressing cells, whereas their levels were elevated in cells transfected with miR-630 inhibitors. Conversely, up-regulation of miR-630 caused a significant increase in E-Cadherin, whereas inhibition of miR-630 resulted in a significant decrease in E-Cadherin (Figure 4D, 4E). These results imply that miR-630 suppresses Slug and in turn attenuates the expression of EMT-related genes.

### MiR-630 attenuates migration, invasion, and EMT in HCC cells by targeting Slug

**Figure 5:**
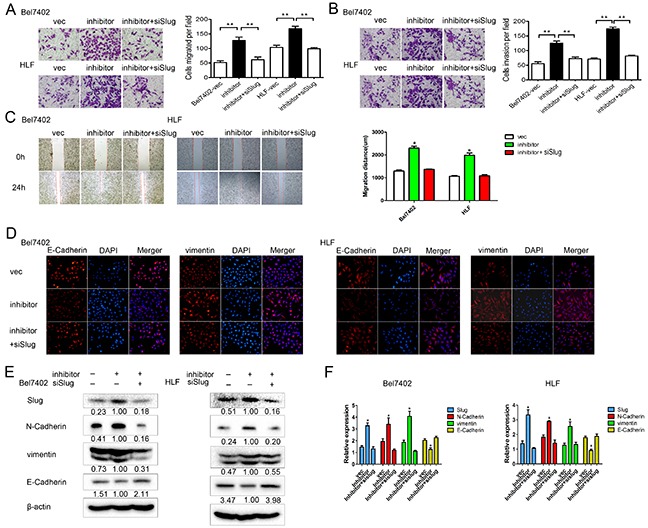
miR-630 exerts its functions by inhibiting Slug in HCC **A.** Results of the migration assay for Bel7402 and HLF cells transfected with miR-630 inhibitor + siRNA against Slug, miR-630 inhibitor only or vector only. Representative photographs were taken at a magnification of ×100. **B.** Results of the invasion assay for Bel7402 and HLF cells transfected with miR-630 inhibitor + siRNA against Slug, miR-630 inhibitor only or vector only. Representative photographs were taken at a ×100 magnification. **C.** The wound healing assay was used to assess HCC cell migration. A confluent monolayer of HCC cell was transfected with vector, miR-630 inhibitor only or miR-630 inhibitor + siRNA against Slug. Photographs were taken immediately (0 h) after the scratch was made and at 24 h after wounding. The amount of wound closure that occurred after 24 h was quantified. **D.** Immunofluorescent staining for the expression of E-Cadherin and vimentin (red) in cells transfected with miR-630 inhibitor + siRNA against Slug, miR-630 inhibitor only or vector only. DAPI was used to show the location of the nuclei (blue). Representative photographs of immunofluorescence were taken at a ×200 magnification. **E.** Western blot analysis of the expression levels of Slug, E-Cadherin, N-Cadherin, vimentin for miR-630 inhibitor + siRNA against Slug, miR-630 inhibitor only or vector only. β-actin was used as a loading control for each sample. **F.** The gene expression levels of Slug, E-Cadherin, N-Cadherin, vimentin, were measured by qRT-PCR for samples transfected with miR-630 inhibitor + siRNA against Slug, miR-630 inhibitor only or vector only. In each sample, gene expression was normalized to GAPDH expression.

To examine whether miR-630 abrogated migration, invasion, and EMT by targeting Slug, we knocked down Slug using siRNA in Bel7402 and HLF cells transfected with miR-630 inhibitors. Transwell assays showed the knockdown of Slug decreased the cell migration and invasion induced by miR-630 inhibitors (Figure 5A, 5B). Similarly, the wound healing assay also revealed that the reduction of Slug rescued the effect of miR-630 inhibitors on the migration (Figure 5C). Moreover, immunofluorescent staining consistently showed the down-regulation of Slug partly suppressed vimentin and increased E-Cadherin initiated by miR-630 inhibitors (Figure 5D). The levels of Slug, N-Cadherin, and vimentin were increased after transfected with miR-630 inhibitors, whereas co-transfection with siRNA against Slug reversed these effects (Figure 5E, 5F). We verified this result by repeating this experiment using a different siRNA sequence against Slug (Supplementary Figure S2). In summary, these data indicate that miR-630 might suppress migration and invasion by inhibiting EMT. Slug serves as a target of miR-630, contributing to the repression effect of miR-630 on metastasis.

### TGF-β represses miR-630 transcription through activation of the ERK/SP1 and JNK/c-Jun pathways in HCC

**Figure 6:**
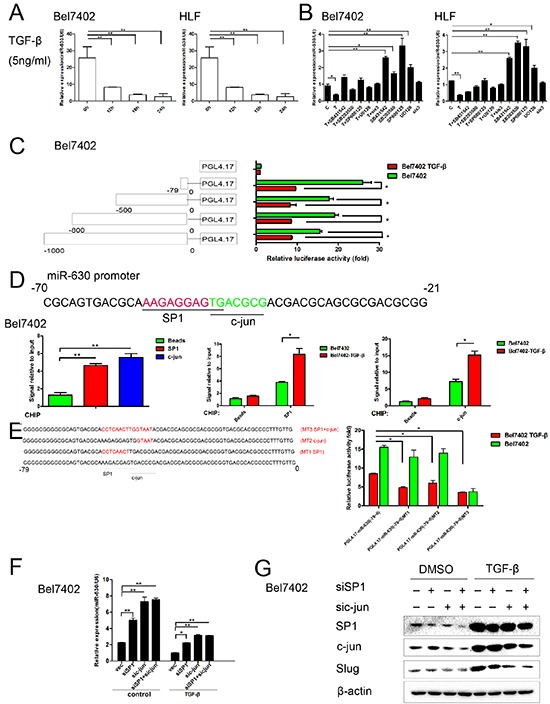
TGF-β represses miR-630 transcription through the activation of the Erk/SP1 and JNK/c-Jun pathway **A.** Cells were treated with 5 ng/mL of TGF-β for 24 h, and the expression level of miR-360 was assessed using RT-PCR. Statistically significant differences are indicated as follows: **P<0.01 compared with the untreated Bel7402 cells. **B.** miR-630 levels after treatment with TGF-β (T) and the indicated inhibitors for 24 h. **C.** A schematic representation of various miR-630 promoter reporters (left). The relative luciferase activity in Bel7402 cells were transfected with various miR-630 promoter reporters or the pGL4.17-basic vector and were either exposed to TGF-β or not (right). The relative luciferase activity was plotted as the mean ± SEM of three independent experiments. **D.** A schematic representation of the 50-bp region of a human miR-630 promoter (−70 to −21 bp). The underlined portions indicate potential binding sites (top). Bel7402 cells were subjected to a quantitative ChIP (qChIP) assay with SP1 or c-Jun antibodies. TGF-β treated and untreated Bel7402 cells were subjected to a ChIP assay with mixed SP1 and c-Jun antibodies (bottom). To control for non-specific genomic DNA binding, beads incubated with DNA extract were used. Statistically significant (P<0.05) results are indicated with an * and (P<0.01) results are indicated with an **. **E.** A schematic representation of the 79-bp region (−79 to 0 bp) of the human miR-630 promoter. Underlined regions indicate the SP1, c-Jun-binding site and mutated SP1, c-Jun-binding site (left). TGF-β treated and untreated Bel7402 cells transfected with indicated promoter reporters and the relative luciferase activity was measured (bottom). **F.** The expression level of miR-630 was measured by qRT-PCR in cells transfected with siSP1, sic-Jun or siSP1+sic-Jun. **G.** Slug expression was measured using the western blot assay in cells transfected with siSP1, sic-Jun or siSP1+sic-Jun. β-actin was included as a loading control for each sample.

Several studies have reported that TGF-β induces Slug transcription [20]. To investigate whether TGF-β represses miR-630 expression, we first examined the levels of miR-630 in TGF-β-treated Bel7402 and HLF cells. Notably, TGF-β treatment resulted in a significant decrease in miR-630 expression in a time-dependent manner (Figure 6A). To determine which signaling pathway activated by TGF-β exerted this effect, we treated Bel7402 and HLF cell with the following kinase inhibitors: SB431542, (a receptor type I TGF-β (TβRI, ALK5) serine/threonine kinase inhibitor), SB203580 (a p38 MAPK inhibitor), SP600125 (a JNK inhibitor), U0126 (a MEK1/2 inhibitor that suppresses Erk signaling) and SIS3 (a SMAD3 inhibitor). The repression of miR-630 by TGF-β was reversed by treatment with U0126, SB203580, SP600125and SB431542 kinase inhibitors but not SIS3 kinase inhibitor (Figure 6B). Western blot analysis confirmed that TGF-β activated the non-SMAD dependent signaling pathways in HCC cell lines (Supplementary Figure S3). To further determine how miR-630 is transcriptionally regulated by TGF-β, representative regions, covering 1.0-kb upstream of the transcriptional initiation site of the miR-630, were investigated using NCBI (http://www.ncbi.nlm.nih.gov/pubmed/) and Jaspar (http://jaspar.genereg.net/) databases. We found that the 1.0-kb region upstream of the transcriptional initiation site of miR-630 contained binding sites for the following TGF-β/non-SMAD dependent pathway transcription factors: ELK-1, CREB1, SP1 and c-Jun (Supplementary Figure S4). Moreover, some reports have shown that miR-630 is located in exon 14 of the ARIH1 gene [18]. To identify the response element essential for the down-regulation of miR-630 promoter activity in response to TGF-β, we introduced a series of miR-630 promoter reporter constructs into Bel7402 cells. The results showed that the region of the miR-630 promoter from −79 to 0 bp was critical for TGF-β-mediated transcriptional repression of miR-630 expression (Figure 6C). Additionally, chromatin immunoprecipitation assays revealed that, in response to TGF-β, SP1 and c-Jun bind the upstream promoter region (-79-0) of the miR-630 gene (Figure 6D). Furthermore, using SP1, c-Jun and SP1+c-Jun mutant constructs, we identified these response elements participated in the TGF-β-repression of miR-630 promoter activity (Figure 6E). Next, we knocked down SP1 and c-Jun using siRNA in HCC cells to determine whether the activation of the ERK/SP1 and JNK/c-Jun signaling pathways by TGF-β is responsible for the repression of miR-630 transcription. Consistently, RT-PCR analysis confirmed the functional role of SP1 and c-Jun in down-regulating miR-630 expression in Bel7402 cells. Knockdown of SP1 or c-Jun could up-regulate miR-630, whereas co-treatment with TGF-β reversed this effect (Figure 6F). Furthermore, the reduction of SP1 and c-Jun in Bel7402 cells repressed the ability of TGF-β to induce Slug expression (Figure 6G). We repeated this experiment using different siRNA sequences to knock down c-Jun and SP1 (Supplementary Figure S5). These results suggest that the Erk/SP1 and JNK/c-Jun signaling pathways exert a critical role for TGF-β in down-regulating miR-630 transcription.

### MiR-630 partly reverses the effects initiated by TGF-β

**Figure 7:**
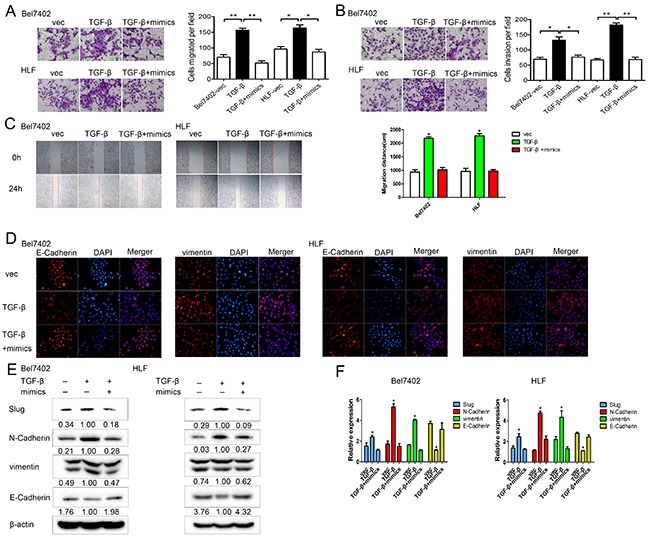
In TGF-β treated HCC cells, miR-630 restores motility and EMT **A.** Migration and **B.** invasion assay for Bel7402 and HLF cells after treatment with either miR-630 mimics + TGF-β, TGF-β only or vector only. Representative photographs were taken at a ×100 magnification. **C.** The wound healing assay was used to assess HCC cell migration. A confluent monolayer of HCC cell was transfected with vector only, TGF-β only or miR-630 mimics + TGF-β. Photographs were taken immediately (0 h) after the scratch was made and at 24 h after wounding. The amount of wound closure that occurred after 24 h was quantified. **D.** Immunofluorescent staining for the expression of E-Cadherin and vimentin (red) in cells treated with miR-630 mimics + TGF-β, TGF-β only or vector only. DAPI was used to show the location of the nuclei (blue). Representative photographs of immunofluorescence were taken at a ×200 magnification. **E.** The expression levels of Slug, E-Cadherin, N-Cadherin and vimentin as measured by western blot in cells treated with TGF-β+miR-630 mimics, TGF-β only or vector only. β-actin was used as a loading control for each sample. **F.** The expression levels of Slug, E-Cadherin, N-Cadherin, vimentin as measured by qRT-PCR in cells treated with TGF-β+miR-630 mimics, TGF-β only or vector only. Gene expression was normalized to GAPDH expression for each sample.

To investigate whether miR-630 reversed the effects induced by TGF-β in HCC, we performed gain-of-function analyses in TGF-β treated cells by up-regulating miR-630 expression. Transwell assays showed that the ectopic expression of miR-630 significantly antagonized the TGF-β-induced migration and invasion of Bel7402 and HLF cells (Figure 7A, 7B). Similarly, the wound healing assay also confirmed that the overexpression of miR-630 suppressed the TGF-β-induced migration (Figure 7C). Furthermore, immunofluorescence staining showed that miR-630 overexpression reversed the TGF-β-mediated up-regulation of vimentin and down-regulation of E-Cadherin (Figure 7D). In addition, E-Cadherin was increased, while Slug, N-Cadherin, and vimentin were decreased after transfection with miR-630 in TGF-β-treated HCC cells (Figure 7E, 7F). Collectively, these data suggest that miR-630 attenuated the TGF-β-induced EMT of HCC cells.

## DISCUSSION

Several studies have shown that miRNAs are key components of biological pathways, including oncogenesis [21–27]. In this study of HCC tissues, we found that miR-630 expression was inversely correlated with metastasis, Edmondson-Steiner tumor grade, TNM stage and BCLC stage. Moreover, low miR-630 expression indicated a higher recurrence rate and shorter overall survival than those with high miR-630 expression. These results indicate that miR-630 may inhibit HCC metastasis, but not tumor size, thereby serving as a prognostic marker for HCC metastasis.

To further investigate the effects of miR-630 on HCC cells, we performed gain-of- and loss-of-function experiments. Our data showed that up-regulation of miR-630 suppressed HCC migration, invasion, and EMT, but not proliferation *in vitro*. Knockdown of miR-630 increased intra-hepatic and lung metastasis, but not tumorigenicity *in vivo*. In our study, the pro-oncogene Slug was identified as a direct and functional target of miR-630 in HCC. Slug, a nuclear transcription factor, represses the transcription of E-Cadherin mRNA by binding to the E-box element of the E-Cadherin promoter [28–30]. The down-regulation of E-Cadherin is responsible for the mesenchymal phenotype observed during EMT [31, 32]. The following results identified Slug as a functional target of miR-630 in HCC cells: (1) Slug expression in HCC tissues was inversely correlated with miR-630 expression; (2) ectopic expression of miR-630 reduced Slug levels significantly in HCC cells, whereas inhibition of miR-630 contributed the opposite effect (3) ectopic expression of miR-630 reduced the activity of a luciferase reporter fused to the WT, but not the MUT 3′-UTR of Slug. Yet, the miR-630 inhibitor group also had an opposite effect compared with the miR-630 mimics group. (4) Down-regulation of Slug abrogated the migration- and invasion-promoting effects induced by the miR-630 inhibitors. Overall, these results demonstrated that miR-630 exerts its anti-metastatic functions by targeting Slug.

Proliferation and metastasis are two of the most important hallmarks of malignancy and major causes of cancer-related death. Previous reports have shown that miR-630 decreases apoptotic cell death by blocking PTEN expression but inhibits proliferation in breast and lung cancers [9–11]. In our study, miR-630 had no effect on proliferation. It is probable that miR-630 might target a set of mRNAs in response to cell cycle and apoptosis, thereby maintaining the proliferation balance.

TGF-β has been regarded as a key driver for EMT in different cancer types [32, 33]. Recent studies have shown that miRNAs regulated downstream molecules of TGF-β to modulate EMT. MiR-125b inhibited EMT by targeting SMAD2 in HCC [34]. Hongping Xia et al. suggested that the miR-106b-25 cluster activated TGF-β signaling by targeting SMAD7 to induce EMT [35]. Kogure T et al. demonstrated that miRNA-29a was involved in the epigenetic regulation of TGF-β-induced EMT in hepatocellular carcinoma [36]. Jia-Yuan Huang et al. reported that miR-451 functioned as an EMT inhibitor by targeting the c-Myc/Erk1/2 axis [37]. Previous studies have reported that TGF-β promoted EMT by directly inducing Slug transcription in HCC [38]. In this study, we showed that the molecular mechanism by which TGF-β promoted EMT was by suppressing the miR-630/Slug axis. Moreover, we found that the 1.0-kb region upstream of the transcriptional initiation site of the miR-630 was inhibited by TGF-β via Erk/SP1 and JNK/c-Jun signaling pathway. We identified response elements of SP1 and c-Jun on the miR-630 promoter. Knockdown experiments demonstrated the critical role of SP1 and c-Jun for down-regulating miR-630 in the absence of TGF-β. However, the data also showed that the repression of SP1 or c-Jun could not completely reverse the TGF-β-mediated miR-630 inhibition in Bel7402 cells, which suggests that TGF-β may activate the expression of SP1 and c-Jun (Figure 6G). In addition, we verified that up-regulation of miR-630, exerting an opposite effect of TGF-β, restored the motility, morphology, and the EMT-related gene expression of HCC cells treated with TGF-β.

Interestingly, the mechanism by which TGF-β/P38 reduces miR-630 expression in HCC remains uninvestigated. A database, Jaspar (http://jaspar.genereg.net/), search for known sequences indicated that P38 signaling pathway may interact with the 1.0-kb region upstream of the transcriptional initiation site of the miR-630. Yet, the luciferase reporter assay indicated that miR-630 promoter from −79 to 0 bp was critical for TGF-β-mediated transcriptional inhibition of miR-630, in which there was no P38-binding element. These data suggest that the binding sites for TGF-β/P38 pathway axis may be within several-kbs upstream of the transcriptional initiation site of the miR-630.

**Figure 8:**
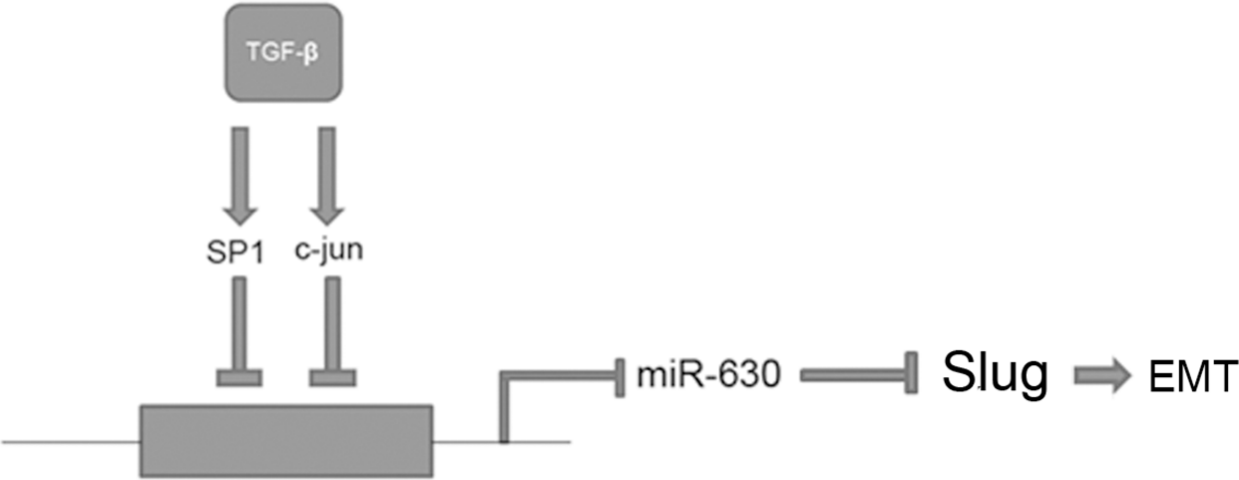
A schematic representation of the major molecular mechanism by which TGF-β promotes EMT in HCC TGF-β represses miR-630 by regulating the SP1, c-Jun/ miR-630 signaling axis. Moreover, TGF-β can promote EMT by indirectly up-regulating Slug.

In conclusion, our results demonstrate that miR-630, which could be transcriptionally reduced by the TGF-β-Erk/SP1 and JNK/c-Jun axis, functions as a tumor suppressor by targeting Slug and subsequently suppressing metastasis in HCC (Figure 8). Furthermore, we provide evidence for a potential mechanism whereby TGF-β promotes HCC cell motility and invasiveness through indirect up-regulation of Slug by repressing miR-630 transcription. Thus, this study reveals a novel TGF-β/miR-630/Slug signaling cascade and further validates the importance of miR-630 in preventing HCC metastasis. We conclude that miR-630 may be a useful prognostic indicator after liver resection and a possible target for future therapy.

## MATERIALS AND METHODS

### Cell culture and transfection

Bel7402 and HLF cells were maintained in Dulbecco's modified Eagle's medium supplemented with 10% fetal bovine serum in a humidified atmosphere of 5% CO_2_ maintained at 37°C. Cytokines, kinase inhibitors and other chemicals used in this study are shown in Supporting Information. All small RNA molecules were ordered from RiboBio (China), including miR-630 mimics, mimics negative controls (mimics-NC), miR-630 inhibitor and inhibitor negative controls (inhibitor-NC). The Bel7402 and HLF cells were transiently transfected with the small RNA molecules or vector using the riboFect TM CP Transfection Kit (333T) (Invitrogen) and following the manufacturer's protocol. The Bel7402 cells were infected with GV280-shmiR-630 lentiviral vector and GV320 lentiviral vector according to the manufacturer's protocol. At 24h post infection, the cells were harvested, and RNA expression was quantified by RT-PCR. Small interfering RNA (siRNA) duplexes targeting slug, c-jun, sp1 and scrambled siRNA are shown in Supporting Information.

### RNA isolation and qRT–PCR

TRIzol reagent (Invitrogen, USA) was used to extract the total RNA from tissues and cells according to a modified version of the manufacturer's protocol. The reverse transcription of miRNA and mRNA was completed using a reverse-transcription system kit (TOYOBO, Japan). Briefly, 1 μg of RNA was reverse transcribed into complementary DNA (cDNA) with random primers or a miR-630 specific stem-loop primer for E-Cadherin, N-Cadherin, vimentin, Slug or miR-630 (primers used are shown in Supporting Information). qPCR analysis was performed with a standard SYBR-Green PCR kit (TOYOBO, Japan) according to a Step One system protocol (Applied Biosystems, Foster City, CA) [39]. GAPDH was used as the endogenous control for the detection of mRNA expression levels, and U6 was used as the endogenous control for miRNA expression analysis. Relative quantification analysis was performed using the comparative CT (2^^-ΔΔCT^) method. Each assay was repeated three times, independently of each other.

### Immunofluorescence assay

The cells were transfected with 100 nM of small RNA molecules or miRNA-630 inhibitor and cultured on chamber slides for 48h after which they were stained for E-Cadherin and vimentin expression using immunofluorescence. Briefly, the cells were fixed in 4% paraformaldehyde for 15 min at room temperature, permeabilizedin Triton X-100 solution (0.5% TritonX-100 dissolved in PBS) for 15 min and incubated with a 1:100 dilution of E-Cadherin and vimentin antibodies for 16h. Nuclei were stained for 3 min with DAPI (10 mg/ml). Alexa Fluor 488conjugated anti-mouse IgG and Alexa Flour 555-conjugated anti-rabbit IgG (Beyotime Institute of Biotechnology) were used as a secondary antibody. Antibodies used are shown in Supporting Information.

### Western blot assay

Western blot analysis was performed as previously described [40, 41] Protein expression was quantified by densitometry and normalized to β-actin or β-tubulin expression using Alpha View software. Antibodies used are shown in Supporting Information.

### Immunohistochemistry assay

Peroxide blocking was conducted with 0.3% peroxide in absolute ethanol. Antigen retrieval in the tissue sections was performed in boiling citrate buffer for 10 min. After antigen retrieval, the slides were incubated overnight at 4°C with a 1:100 dilution of the Slug primary antibody (Abcam) and then incubated with a secondary antibody (Dako, Denmark) at 37°C for 1 h. Slides were then washed twice with PBS and then the color reaction was performed using the DAB work solution (Dako). The immunostaining results were assessed and scored independently by two pathologists as described previously [41].

### Chromatin immunoprecipitation assay

For ChIP assays, cells were cultured in 10-cm culture dishes (a 4×10^6^ cells/dish) and were DNA-protein cross-linked in PBS containing 1% formaldehyde at room temperature for 10 min. Subsequently, the ChIP procedure was performed with the Simple ChIP 1 Plus Enzymatic Chromatin IP Kit (Magnetic Beads) (#9005, Cell Signaling Technology, Beverly, MA) according to the manufacturer's instructions. For each ChIP, the precleared lysate and the mixtures were incubated with specific antibodies (Supporting Information) and rotated at 4°C overnight. The Beads alone, incubated with DNA extract was used as a negative control for non-specific genomic DNA binding. The immunocomplexes were pulled down by Protein A Agarose/Salmon Sperm DNA (50% Slurry) and then washed with the Low-Salt Immune Complex Wash Buffer, the High-Salt Immune Complex Wash Buffer, the LiCl Immune Complex Wash Buffer and finally two times with TE Buffer. The bound protein was eluted with elution buffer containing 1% SDS and 0.1MNaHCO_3_. The crosslinks were reversed by heating at 65°C for 4 hours. The DNA was purified by the QIAquick PCR purification Kit (QIAGEN)To detect the amount of immunoprecipitated DNA product, qPCR amplifications were performed using the SYBR Green PCR Master Mix-PLUS (TOYOBO, Osaka, Japan) according to the manufacturer's instructions (Xiang et al., 2012). The qPCR data from the IPs was normalized with the values of the same input sample.

### Statistical analysis

All statistical analyses were performed using SPSS 18.0 statistical software. Overall survival and disease-free survival were calculated using the Kaplan–Meier method and the log-rank test. Continuous data were compared using Student's two-tailed test. Data are represented as the mean ±SEM. Analysis of variance (ANOVA) was performed to determine statistically significant differences. A value of P < 0.05 was considered statistically significant, *, P< 0.05; **, P< 0.01.

## SUPPLEMENTARY MATERIALS AND METHODS




