# Supplementary material for: MicroRNA-630 suppresses tumor metastasis through the TGF-β- miR-630-Slug signaling pathway and correlates inversely with poor prognosis in hepatocellular carcinoma
Source: Oncotarget. 2016 Mar 14;7(16):22674–86. doi: 10.18632/oncotarget.8047 (PMC5008391; doi:10.18632/oncotarget.8047)
Supplement: Supplementary file 1 [file oncotarget-07-22674-s001.pdf]

SUPPLEMENTARY MATERIALS AND METHODS

Patients and specimens

Ninety-seven human liver tumor tissues were obtained between 2010 and 2012 from patients who underwent liver resection surgery at the Hepatic Surgery Center of Tongji Hospital affiliated with the Huazhong University of Science and Technology. The pathology of all of the tissues specimens was verified and all of the specimens were stored at –80°C until used for analysis. Overall survival (OS) was defined as the interval between the date of resection and the date of patient death or last follow-up. For surviving patients, the data were censored at the last follow-up. Time to recurrence (TTR) was defined as the interval between the date of surgery and the date of diagnosis of any type of relapse (intra-hepatic recurrence and extra-hepatic metastasis).The study protocol was approved by the ethics committee of the Huazhong University of Science and Technology and Tongji Hospital and a written informed consent were obtained from all participants involved in this study.

Cell proliferation assay

Two thousand cells per well were plated in 96-well plates and incubated at 37°C. Cell proliferation was assessed at various time points after transfection using the Cell Counting Kit-8 (Beyotime Institute of Biotechnology) according to the manufacturer's instruction. Each assay was repeated three times.

Cell migration and invasion assays

For Transwell cell migration and invasion assays, tumor cells ($2x10^4$) in 0.2 ml of serum-free medium were seeded onto the 8μm insert (upper chamber) of a Transwell plate (Corning Costar, NY, USA). The lower chamber was filled with 0.5 ml of medium containing 10% FBS. For the migration assay, cells were incubated for 24 h post-seeding, after which, the cells remaining in the upper part of the Transwell were removed with a cotton swab. Cells that had migrated to the lower chamber were then stained with 0.5% Crystal Violet dye, and the number of cells per high field (×100) was counted using a Nikon microscope. For cell invasion assays, Transwell chamber inserts were pre-coated with Matrigel (BD Biosciences, NJ, USA). After a 48 h incubation period post-seeding, the number of cells invading the Matrigel-coated insert was counted.

Wound healing assay

Cells (4×10^5 /well) were seeded in 12-well plates, cultured overnight. After forty-eight hours, a 5-mm-wide scratch was made across the cells using a sterile plastic tip, and the cells were washed twice with culture medium. Fresh serum-free medium was then added, and the imaging was performed after 0 h and 24 h using an NIKON Digital ECLIPSEC microscope and a Retiga-4000DC camera. The images were analyzed using Cell Profiler.

Cell counting assay

Cells were digested by trypsin. The number of cells was counted by Cellometer Mini (Nexcelom Bioscience, Massachusetts USA). Each assay was repeated three times.

Luciferase reporter assay

We used the Dual-Luciferase Reporter Assay System (Promega, USA) to verify the precise target of miRNAs and the specific response element that participated in the down-regulation of miR-630 promoter activity. Briefly, $1x10^5$ Bel7402 cells were seeded in 24-well plates. After 24 h, the luciferase reporter plasmid was co-transfected with small RNA molecules or TGF-β using the riboFect TM CP Transfection Kit (333T) (Invitrogen). Firefly and Renilla luciferase activities were measured 24 h post-transfection using the DualGlo Luciferase Assay System (Promega, USA). Firefly luciferase was normalized to Renilla luciferase activity. All experiments were performed three times.

Animal studies

Six-week-old male BALB/c athymic nude mice were housed under specific pathogen free (SPF) conditions and cared for according to the institutional guidelines for animal care. All animal studies conformed to the National Institutes of Health guidelines (NIH publication 86-23 revised 1985) and were approved by the Committee on the Ethics of Animal Experiments of the Tongji Medical College, HUST. The Bel7402-shmiR-630 and NC-transfected

clone cells were harvested and resuspended to 2 × 10^6 cells/ml in serum-free DMEM. For the *in vivo* tumorigenicity assay, we injected 2 × 10^6 cells in a total volume of 100µl subcutaneously into the right flanks of athymic nude mice (n=10). All mice were monitored once every three days and were sacrificed four weeks later. Tumor weight was monitored. For *in vivo* tumor metastasis assays, subcutaneous tumor tissue was minced into small pieces of equal volume (1 mm3) and transplanted into the livers of nude mice (10 mice per group). All mice were monitored once every three days, then sacrificed eight weeks later. Their liver and lung tissues were dissected, fixed and prepared for histological examination.

SUPPLEMENTARY FIGURES AND TABLES

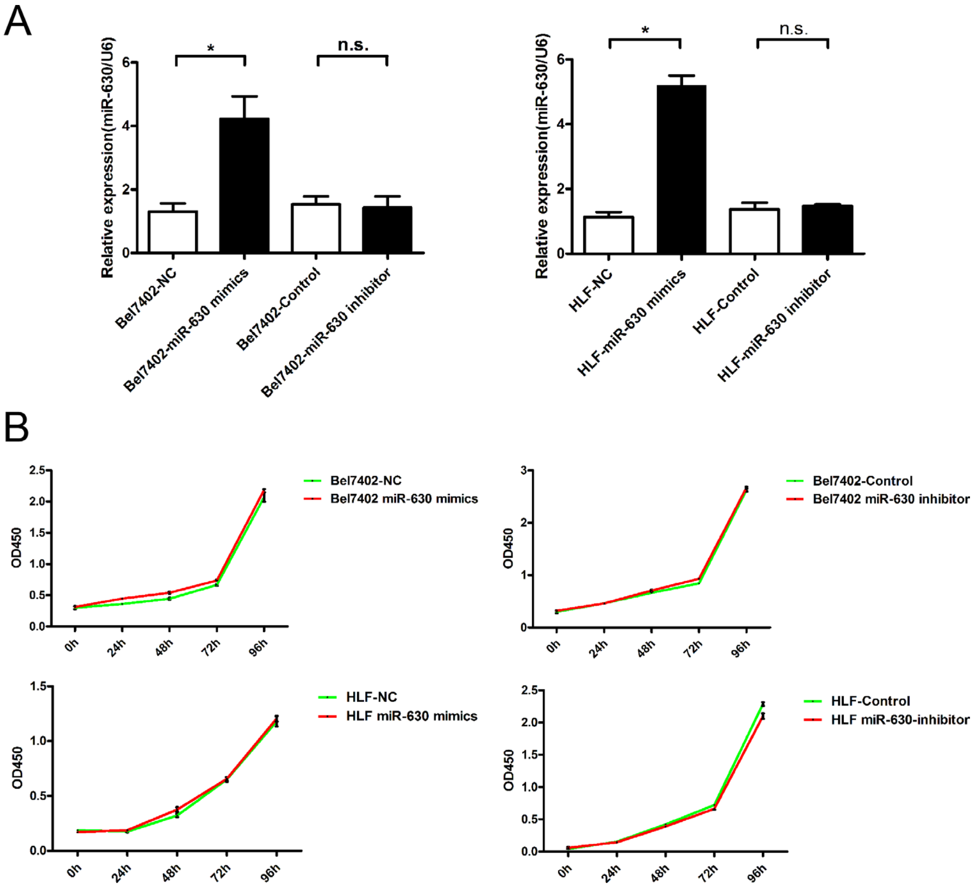

Supplementary Figure S1: Related to Figure 2. MiR-630 has no effect on the growth of Bel7402 and HLF cells. **A.** Bel7402 and HLF cells were transfected with miR-630 mimics or inhibitor. **B.** Indicated HCC cells were subjected to the CCK-8 assay. Comparisons of the miR-630 mimics or inhibitor groups versus the control group were made using relevant statistical test (n=3). * indicates a statistically significant result with $P<0.05$; n.s. indicates no significance.

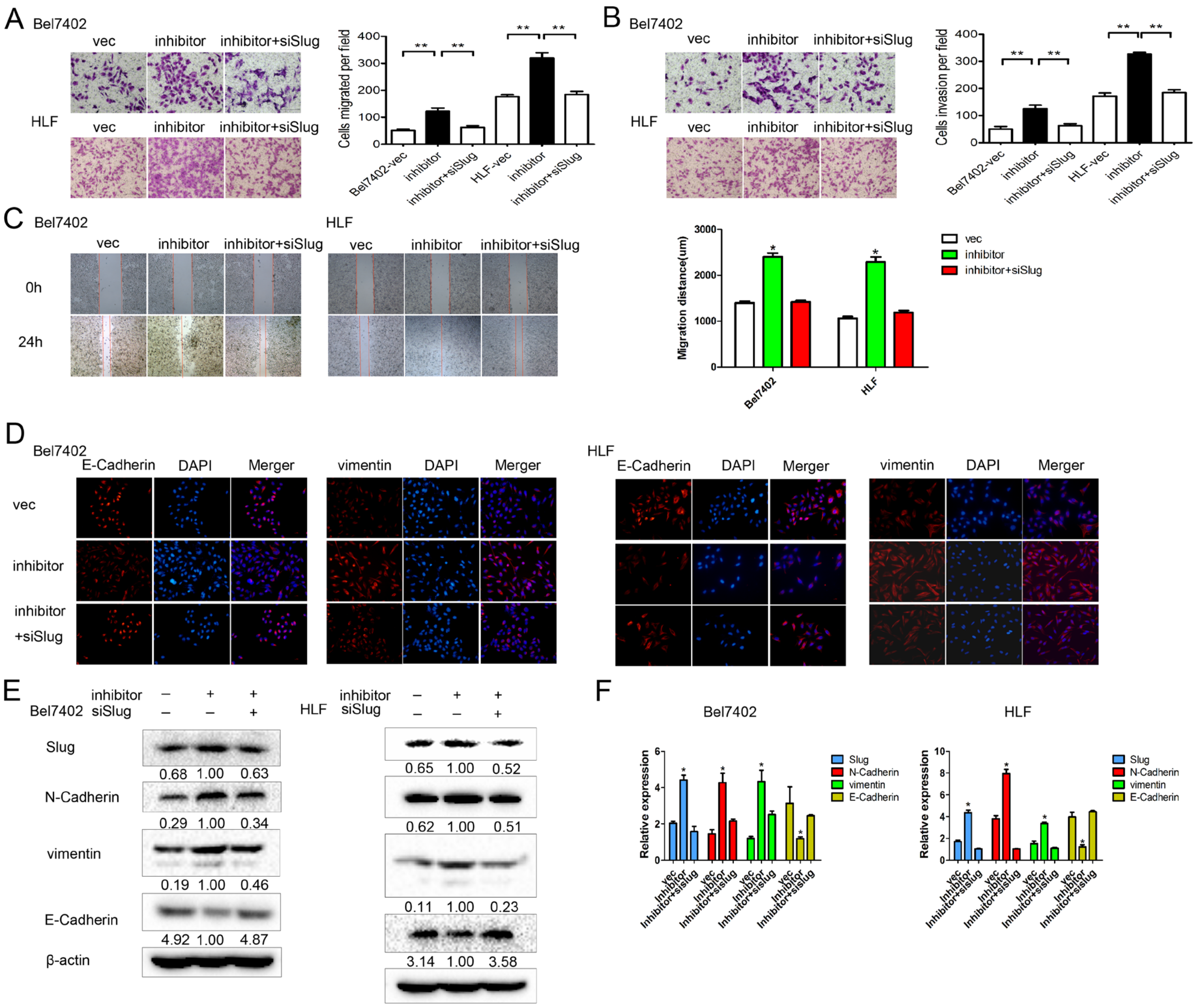

Supplementary Figure S2: Related to Figure 5. Our conclusions were strengthened by using another siRNA targeting Slug.

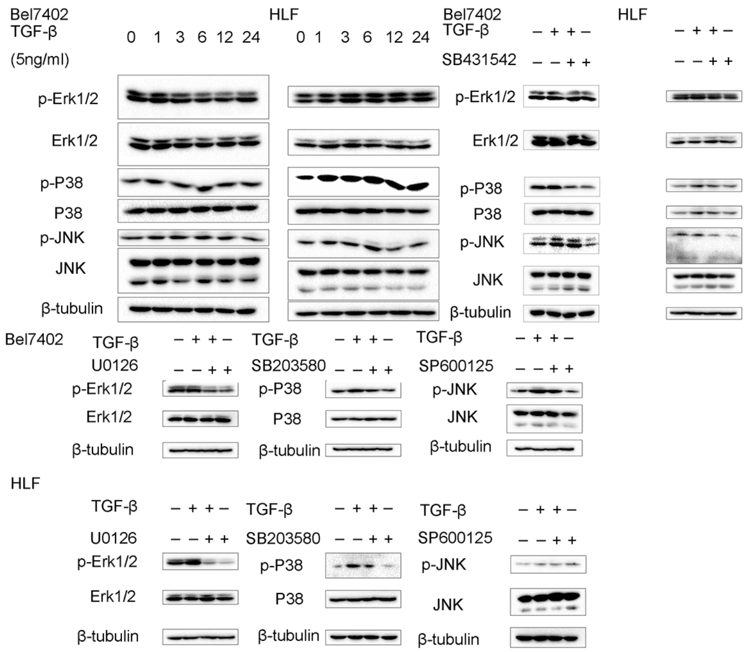

Supplementary Figure S3: Related to Figure 6. Proteins in the JNK, Erk and P38 signaling pathways were measured by Western blot after cells were treated with TGF-β and the indicated inhibitors.

TTGAACTCCTAGCCATTCATCAACACTCAGCTCACATAGCACACTTTCAG
ATGAGATTCAAGCATTATCTCTCTGGCTACATTTGGCCCTTGTAGATATTT
CTATTATTGCCTGTATTTTGTTGAAGCATTCATGTCTTTTATGTACTACATA
GTACATAATATATCTACATTACAAAGCATTTTTATTGATTCATGTTTTGGGG
CATCCAGCGGTTAAAATAGGGCAAGGGTTATGTGGTCTTGGCCAGGTCC
CTTTTCCTTTCTGAGCTTCAGGTTCCTCAGGTATAAAATGGGTACTATGG
CTCCGTGAAGATGAGACAGAATATATGTAAAGCCCTTACACAGTGCTCAA
TTCATGAAAATCATCGCTGTTTACATTGTTTAGGGCCCTCAAGAGGCGCT
CTTGTCAGCGGGGTGGAGGAAATGCCTCTTTAAAATGACGTCATTATAG
GAGACTTAAAAACCACTGCACGTCTGTACAAGTGGACAGAATCACACAG
CTACCTAAAGTGGGTTCACCTGTCATACAGTTTAGAGGTTCCACTTACAA
CAGGTTTCCATCAGTAGCCAAAGCAACAGAAATTTTAGCTTAACGGCTGA
GGGCTTCCGGGATTACCGCAGGGCGGGCGGGAAGCGCCTTTCCGCTCC
GGAGGGAAGGCCGCGTCCCCAAGGGACTGCCTAAGTGGGCTGGATCCC
TATGGCCTGCGGCAGACACCACCACGTAGGCGGTCCAGGGGTCCCTCT
CTCGGTGACTGGTGACCAGGAGGCCGCAATCTCTTCACATAAAAGGTCT
ACGGGTTTCCGCTCGGCCCTCACAGCGGCAGCGGATCGAACCAGGAAA
GCAATTCCCGCTCGGCTCCCCTCGGGTCCCAGGCCCAGGAAGGCCGGG
CCGTGGGGTCTCCCCGGGCTCCACGCAGGGGCGGTGGGGCGGGGCG
CAGTGACGCAAAGAGGAGTGACGCGACGACGCAGCGCGACGCGGTGA
CGCACGCCCCTTTGTTG

Supplementary Figure S4: Related to Figure 6. The TGF-β/non-SMAD dependent pathway transcription factors bind regions of the miR-630 promoter. Representative regions 1.0-kb upstream of the transcription initiation site of the miR-630 is shown. The names of the color coded transcription factor binding sites are as follows: c-Jun, CREB1, ELK-1, SP1 and c-Jun.

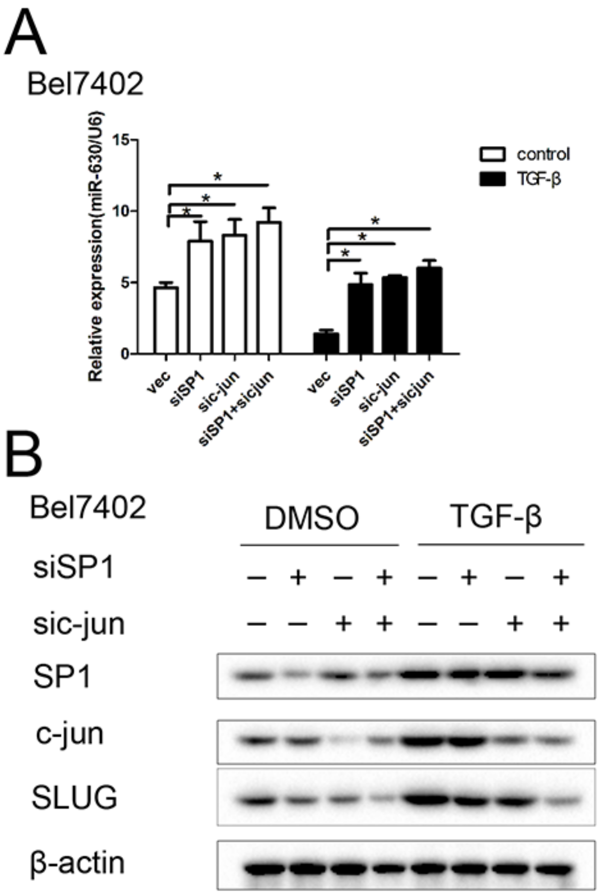

Supplementary Figure S5: Related to Figure 6. Our conclusions were strengthened by using another siRNA targeting c-Jun or SP1.

Supplementary Table S1: Correlations between miR-630 expression level and clinicopathological variables 97 cases of HCC

Clinicopathological variables	Relative miR-630 expression		P value
	Low n=49	High n=48	
Gender			
Male	44	44	1.000
Female	5	4	
Age			
≤50	32	25	0.219
>50	17	23	
AFP (ug/L)			
≤20	4	16	0.003
>20	45	32	
GGT(u/l)			
≤54	16	21	0.300
>54	33	27	
ALT(ng/mL)			
≤75	40	34	0.240
>75	9	14	
HBV			
Negative	6	4	0.740
Positive	43	44	
Cirrhosis			
No	4	8	0.233
Yes	45	40	
Tumor size (cm)			
≤5	17	21	0.409
>5	32	27	
Tumor encapsulation			
None	24	32	0.101
Complete	25	16	
Tumor number			
Single	33	42	0.028
Multiple	16	6	
Vascular invasion			
No	28	39	0.015
Yes	21	9	

(*Continued*)

Clinicopathological variables	Relative miR-630 expression		P value
	Low n=49	High n=48	
Edmondson			
I-II	28	40	0.007
III-IV	21	8	
BCLC stage			
0+A	22	37	0.002
B+C	27	11	

Chi-square test or the Fisher exact test; Statistically significant ($p<0.05$)

Supplementary Table S2: Univariate and multivariate analysis of factors associated with overall survival and disease-free survival of 97 HCC patients

(See Supplementary File 1)
