# Supplementary material for: MicroRNA-630 suppresses tumor metastasis through the TGF-β- miR-630-Slug signaling pathway and correlates inversely with poor prognosis in hepatocellular carcinoma
Source: Oncotarget. 2016 Mar 14;7(16):22674–86. doi: 10.18632/oncotarget.8047 (PMC5008391; doi:10.18632/oncotarget.8047)
Supplement: Supplementary file 2 [file oncotarget-07-22674-s002.docx]

**Table 2: Univariate and multivariate analysis of factors associated with overall survival and disease-free survival of 97 HCC patients.**

|  | **disease-free survival** | |  |  | | | **overall survival** | |  | | | |
| --- | --- | --- | --- | --- | --- | --- | --- | --- | --- | --- | --- | --- |
|  | **univariate analysis** | |  | **multivariate analysis** | |  | **univariate analysis** | |  | **multivariate analysis** | |  |
|  | **HR** | **95%CI** | **p value** | **HR** | **95%CI** | **p value** | **HR** | **95%CI** | **p value** | **HR** | **95%CI** | **p value** |
| **Age(>50 vs ≤50)** | **0.516** | **0.256-1.040** | **0.064** |  | | | **0.778** | **0.310-1.951** | **0.593** |  | | |
| **Gender (male vs female)** | **0.628** | **0.245-1.612** | **0.333** |  |  |  | **0.391** | **0.131-1.171** | **0.093** |  |  |  |
| **Cirrhosis(yes vs no)** | **0.974** | **0.384-2.522** | **0.985** |  |  |  | **1.508** | **0.349-6.513** | **0.582** |  |  |  |
| **HBV(positive vs negative)** | **1.665** | **0.511-5.423** | **0.397** |  |  |  | **1.34** | **0.310-5.793** | **0.695** |  |  |  |
| **Edmondson(III-IV vs I- II)** | **2.434** | **0.744-2.762** | **0.281** |  |  |  | **1.646** | **0.673-4.028** | **0.275** |  |  |  |
| **Serum AFP(>20 vs ≤20ug/L)** | **1.446** | **0.606-3.453** | **0.406** |  |  |  | **1.628** | **0.477-5.559** | **0.437** |  |  |  |
| **Tumor number(multiple vs single)** | **3.273** | **1.680-6.378** | **0** | **1.322** | **0.539-3.243** | **0.542** | **4.04** | **1.676-9.735** | **0.002** | **0.74** | **0.217-2.530** | **0.632** |
| **Tumor size (cm)(>5 vs≤5)** | **2.71** | **1.284-5.719** | **0.009** | **1.532** | **0.661-3.550** | **0.32** | **4.092** | **1.199-13.973** | **0.025** | **1.908** | **0.442-8.229** | **0.386** |
| **Tumor encapsulation( none vs complete)** | **2.786** | **1.454-5.338** | **0.002** | **1.477** | **0.713-3.060** | **0.294** | **2.812** | **1.121-7.052** | **0.028** | **1.37** | **0.494-3.796** | **0.545** |
| **Vascular invasion( yes vs no)** | **3.631** | **1.904-6.923** | **0** | **1.071** | **0.344-3.337** | **0.906** | **3.324** | **1.374-8.042** | **0.008** | **0.498** | **0.130-1.911** | **0.31** |
| **TNM stage(III-IV vs I-II)** | **4.851** | **2.449-9.610** | **0** | **1.769** | **0.358-8.753** | **0.484** | **10.806** | **3.155-37.015** | **0** | **13.857** | **1.184-162.146** | **0.036** |
| **BCLC stage(B+C vs 0+A)** | **5.001** | **2.517-9.937** | **0** | **1.668** | **0.280-9.949** | **0.575** | **8.065** | **2.687-24.208** | **0** | **0.999** | **0.088-11-332** | **0.999** |
| **miR-630 expression(high vs low)** | **0.42** | **0.215-0.818** | **0.011** | **0.662** | **0.325-1.347** | **0.225** | **0.379** | **0.145-0.987** | **0.047** | **0.705** | **0.259-1.918** | **0.494** |

Statistically significant (P<0.05)

The primers use for PCR

| Gene  name |  | Primer Sequence |
| --- | --- | --- |
| miR-630  U6  Slug | F  R  F  R  F | 5’-AACTTA ACATCATGCTACCT-3’  5’-ATATAGTTAAGA ACTACCTT-3’  5’-CTCGCTTCGGCAGCACA-3’  5’-AACGCTTCACGAATTTGCGT-3’  5’- GGGGAGAAGCCTTTTTCTTG -3’ |
|  | R | 5’- TCCTCATGTTTGTGCAGGAG -3’ |
| E-Cadherin | F | 5’-CTGAGAACGACTGGCTAACG-3’ |
|  | R | 5’-TTCACATCCAGCACATCC-3’ |
| N-Cadherin | F | 5’-GGACGCCGAGCCCCAGTATC-3’ |
|  | R | 5’-CCCCCAGTCGTTCAGGTAATCA-3’ |
| vimentin  GAPDH  qChIP | F | 5’-TTGAACGCAAAGTGGAATC-3’ |
|  | R  F  R  F  R | 5’-AGGTCAGGCTTGGAAACA-3’  5’-TGGGTGTGAACCATGAGAAGT-3’  5’-TGAGTCCTTCCACGATACCAA -3’  5’-GCGGCAGACACCACCAC-3  5’-GGCCGCTGCTATCGCTACTGAG-3’ |

Antibodies used in this study.

| Antigens | Manufacturers | Application |
| --- | --- | --- |
| E-cadherin | 610181, BD Transduction Laboratories, San  Jose, CA, USA | 1:5000 for WB  1:300 for IF |
| N-cadherin | 18-0224, Life Technologies, Carlsbad, CA, USA | 1:1000 for WB |
| Vimentin | #5741, Cell Signaling Technology, Beverly, MA, USA | 1:1000 for WB  1:100 for IF |
| Phospho-p44/42 MAPK (Erk1/2) (Thr202/Tyr204) | #4370, Cell Signaling Technology, Beverly, MA, USA | 1:10,00 for WB |
| p44/42 MAPK (Erk1/2) | #4695, Cell Signaling Technology, Beverly, MA, USA | 1:10,00 for WB |
| Phospho-p38 | #4511, Cell Signaling Technology, Beverly, MA, USA | 1:1000 for WB |
| p38 | #8690, Cell Signaling Technology, Beverly, MA, USA | 1:1000 for WB |
| Phospho-SAPK/JNK | # 4668S, Cell Signaling Technology, Beverly, MA, USA | 1:1000 for WB |
| SAPK/JNK Antibody | #9252, Cell Signaling Technology, Beverly, MA, USA | 1:1000 for WB |
| Slug (C19G7) | #9585, Cell Signaling Technology, Beverly, MA, USA | 1:1000 for WB |
| SLUG | Abcam ,ab27568 | 1:100 for IHC |
| c-Jun (60A8) | #9165, Cell Signaling Technology, Beverly, MA, USA | 1:1000 for WB  1:50 for CHIP |
| SP1 (D4C3) | #9389, Cell Signaling Technology, Beverly, MA, USA | 1:1000 for WB  1:100 for CHIP |
| β-actin | sc-47778, Santa Cruz Biotechnology, Santa Cruz, CA, USA | 1:30,00 for WB |
| GAPDH | KC-5G4, KangChen Bio-tech, Shanghai, China. | 1:10,000 for WB |
| β-Tubulin | M30109, Abmart, Shanghai, China. | 1:5000 for WB |
| Alexa Flour 555-conjugated anti-rabbit IgG | Beyotime Institute of Biotechnology, Jiangsu, China | 1:500 for IF |
| Alexa Flour 488-conjugated anti-mouse IgG | Beyotime Institute of Biotechnology, Jiangsu, China | 1:500 for IF |
| Horseradish peroxidase (HRP) conjugated anti-rabbit IgG | Jackson ImmunoResearch Laboratories, Inc. West Grove, PA, USA | 1:5000 for WB |
| HRP conjugated anti-mouse IgG | Jackson ImmunoResearch Laboratories, Inc. West Grove, PA, USA | 1:5000 for WB |
| Secondary antibody | Envision kit (HRP, rabbit/mouse, DAB+), DAKO | Ready-to-use for IHC |

Abbreviations:WB: Western Blot IF:immnuoflurorescence;

IHC:immunohistochemistryChIP: chromatin immunoprecipitation

Sequence of the siRNA Duplexes and the miRNA-inhibition

| siRNA | Sequence |
| --- | --- |
| si-h-SNAI2_001 | 5‘ GGAGCAUACAGCCCCAUCA dTdT 3‘  3‘ dTdT CCUCGUAUGUCGGGGUAGU 5‘ |
| si-h-SNAI2_002 | 5‘ CUUCAAGGACACAUUAGAA dTdT 3‘  3‘ dTdT GAAGUUCCUGUGUAAUCUU 5‘ |
| si-h-SNAI2_003 | 5‘ GCAUUUGCAGACAGGUCAA dTdT 3‘  3‘ dTdT CGUAAACGUCUGUCCAGUU 5‘ |
| Si-h-SP1 | 5‘ CCAACAGAUUAUCACAAAU dTdT 3‘  3‘ dTdT GGUUGUCUAAUAGUGUUUA 5‘ |
| si-sp1_2 | 5‘ GCCAAUAGCUACUCAACUA dTdT 3‘  3‘ dTdT CGGUUAUCGAUGAGUUGAU 5‘ |
| si-sp1_3 | 5‘ GCCAAUAGCUACUCAACUA dTdT 3‘  3‘ dTdT CGGUUAUCGAUGAGUUGAU 5‘ |
| si-c-jun_1 | 5‘ GACCUUAUGGCUACAGUAA dTdT 3‘  3‘ dTdT CUGGAAUACCGAUGUCAUU 5‘ |
| si-c-jun_2 | 5‘ GGCACAGCUUAAACAGAAA dTdT 3‘  3‘ dTdT CCGUGUCGAAUUUGUCUUU 5‘ |
| si-c-jun_3 | 5‘ CGCAGCAGUUGCAAACAUU dTdT 3‘  3‘ dTdT GCGUCGUCAACGUUUGUAA 5‘ |
| Scramble-siRNA | 5‘TTCTCCGAACGTGTCACGT3‘  3‘caACGTGACACGTTCGGAGAA5‘ |
| hsa-miR-630-inhibition(9714-1) | 5‘AATTCAAAAAAGTATTCTGTACCAGGGAAGGT3‘  5‘Ccgg ACCTTCCCTGGTACAGAATACT TTTTTg3‘ |
| Scramble-vector | 5‘TTCTCCGAACGTGTCACGT3‘  5‘ACGTGACACGTTCGGAGAA3‘ |

Cytokines, kinase inhibitors and other chemicals

| Chemicals | Manufacturers |
| --- | --- |
| Recombinant human TGF-β  Receptor II/Fc Chimera | 10358-H03H, Sino Biological Inc., Beijing, China |
| SB431542 | 301836-41-9, Cayman, Ann Arbor, MI, USA |
| SB203580 | S1863, Beyotime Institute of Biotechnology,  Shanghai, China |
| SP600125 | 1496, Tocris, Bristol, United Kingdom |
| U0126 | 1144, Tocris, Bristol, United Kingdom |
| SIS3 | S0447, Sigma-Aldrich, St. Louis, MO, USA |
